# The Bartter-Gitelman Spectrum: 50-Year Follow-up With Revision of Diagnosis After Whole-Genome Sequencing

**DOI:** 10.1210/jendso/bvac079

**Published:** 2022-05-15

**Authors:** Mark Stevenson, Alistair T Pagnamenta, Heather G Mack, Judith Savige, Edoardo Giacopuzzi, Kate E Lines, Jenny C Taylor, Rajesh V Thakker

**Affiliations:** Academic Endocrine Unit, Oxford Centre for Diabetes, Endocrinology & Metabolism (OCDEM), Churchill Hospital, University of Oxford, Oxford OX3 7LJ, UK; National Institute for Health Research Oxford Biomedical Research Centre, Oxford, OX3 7LJ, UK; National Institute for Health Research Oxford Biomedical Research Centre, Oxford, OX3 7LJ, UK; Wellcome Centre for Human Genetics, University of Oxford, Oxford, OX3 7BN, UK; Department of Surgery (Ophthalmology), University of Melbourne, Parkville, Victoria, 3002, Australia; The University of Melbourne Department of Medicine (Melbourne Health) and Northern Health, Epping, Victoria, 3050, Australia; National Institute for Health Research Oxford Biomedical Research Centre, Oxford, OX3 7LJ, UK; Wellcome Centre for Human Genetics, University of Oxford, Oxford, OX3 7BN, UK; Academic Endocrine Unit, Oxford Centre for Diabetes, Endocrinology & Metabolism (OCDEM), Churchill Hospital, University of Oxford, Oxford OX3 7LJ, UK; National Institute for Health Research Oxford Biomedical Research Centre, Oxford, OX3 7LJ, UK; National Institute for Health Research Oxford Biomedical Research Centre, Oxford, OX3 7LJ, UK; Wellcome Centre for Human Genetics, University of Oxford, Oxford, OX3 7BN, UK; Academic Endocrine Unit, Oxford Centre for Diabetes, Endocrinology & Metabolism (OCDEM), Churchill Hospital, University of Oxford, Oxford OX3 7LJ, UK; National Institute for Health Research Oxford Biomedical Research Centre, Oxford, OX3 7LJ, UK

**Keywords:** kidney, salt-wasting, ion channels, transporters, CLC-Kb

## Abstract

Bartter syndrome (BS) and Gitelman syndrome (GS) are renal tubular disorders affecting sodium, potassium, and chloride reabsorption. Clinical features include muscle cramps and weakness, in association with hypokalemia, hypochloremic metabolic alkalosis, and hyperreninemic hyperaldosteronism. Hypomagnesemia and hypocalciuria are typical of GS, while juxtaglomerular hyperplasia is characteristic of BS. GS is due to *SLC12A3* variants, whereas BS is due to variants in *SLC12A1*, *KCNJ1*, *CLCNKA*, *CLCNKB*, *BSND*, *MAGED2*, or *CASR*. We had the opportunity to follow up one of the first reported cases of a salt-wasting tubulopathy, who based on clinical features was diagnosed with GS. The patient had presented at age 10 years with tetany precipitated by vomiting or diarrhea. She had hypokalemia, a hypochloremic metabolic alkalosis, hyponatremia, mild hypercalcemia, and normomagnesemia, and subsequently developed hypocalciuria and hypomagnesemia. A renal biopsy showed no evidence for juxtaglomerular hyperplasia. She developed chronic kidney failure at age 55 years, and ocular sclerochoroidal calcification, associated with BS and GS, at older than 65 years. Our aim was therefore to establish the genetic diagnosis in this patient using whole-genome sequencing (WGS). Leukocyte DNA was used for WGS analysis, and this revealed a homozygous c.226C > T (p.Arg76Ter) nonsense *CLCNKB* mutation, thereby establishing a diagnosis of BS type-3. WGS also identified 2 greater than 5-Mb regions of homozygosity that suggested likely mutational heterozygosity in her parents, who originated from a Greek island with fewer than 1500 inhabitants and may therefore have shared a common ancestor. Our results demonstrate the utility of WGS in establishing the correct diagnosis in renal tubular disorders with overlapping phenotypes.

Bartter syndrome (BS) and Gitelman syndrome (GS) are salt-wasting renal tubular disorders affecting the excretion and reabsorption of electrolytes such as sodium, potassium, and chloride [1, 2]. Originally considered genotypically and phenotypically distinct, evidence now suggests BS and GS represent a spectrum of disease caused by mutations in different genes encoding proteins involved in chloride reabsorption at different nephron locations [3-5]. Common abnormal metabolic characteristics of BS and GS include hypokalemia with a hypochloremic metabolic alkalosis, and normal or low blood pressure despite hyperreninemia and hyperaldosteronism [6]. Urinary calcium excretion may be differentiating, such that hypocalciuria is typical of GS, and hypercalciuria typical for BS, while hypomagnesemia, which was considered a GS-specific feature, has also been reported in some BS patients [7, 8]. Hyperplasia of the juxtaglomerular apparatus is considered characteristic of BS [6].

BS, which represents a group of genetically heterogeneous disorders with 7 subtypes reported, and GS are caused by variants in genes encoding ion transporters or channels most commonly causing loss of function (Table 1). BS type 1 (MIM: 601678) affects approximately 25% of patients and is associated with mutations of *SLC12A1* (solute carrier family 12 member-1) encoding NKCC2 (sodium-potassium-2 chloride cotransporter); BS type 2 (MIM: 241200) affects 10% to 25% of patients and is due to mutations of *KCNJ1* (potassium channel, inwardly rectifying, subfamily-J, member-1) encoding K_ir_1.1 (ATP-dependent potassium channel; also referred to as renal outer medullary channel); BS type 3 (MIM: 607364) affects approximately 20% to 30% of patients and is due to mutations of *CLCNKB* (chloride channel, kidney-B) encoding CLC-Kb (voltage-gated chloride channel-Kb); BS type 4A (MIM: 602522) affects less than 10% of patients and is due to mutations of *BSND* (Barttin CLCNK-type accessory subunit-beta) encoding an essential subunit for voltage-gated chloride channel-Ka (CLC-Ka) and CLC-Kb; BS type 4B (MIM: 613090) affects less than 5% of patients who also have sensorineural deafness due to mutations of both *CLCNKA* and *CLCNKB* encoding CLC-Ka and CLC-Kb respectively; BS type 5 (MIM: 300971), which occurs as a transient fetal form in approximately 10% of BS patients, is due to mutations of *MAGED2* (melanoma-associated antigen family member-D2); and BS occurring in association with autosomal dominant hypocalcemia (ADH) (MIM: 601198), formerly referred to as BS type 5, results from gain-of-function mutations of *CASR* (calcium-sensing receptor) [4, 5, 9-12]. GS in approximately 80% to 95% of patients is associated with mutations in *SLC12A3* encoding NCCT (thiazide-sensitive Na-Cl cotransporter) (MIM: 263800) [11, 13], and the remaining 5% to 20% of patients with GS-like syndromes may have pathogenic variants in *KCNJ10* (KCNJ member-10) encoding K_ir_4.1, *FXYD2* (FXYD-domain containing ion transport regulator-2) encoding sodium/potassium-transporting ATPase subunit-gamma, *HNF1B* (hepatocyte nuclear factor-1 homeobox-B), or mitochondrial DNA [14-20]. For GS and all BS subtypes the mode of inheritance is monogenic autosomal recessive except those associated with mitochondrial DNA mutations; BS type 4B, which is digenic recessive; BS type 5, which is X-linked recessive; and BS with ADH, or GS-like with *FXYD2* or *HNF1B*, which are autosomal dominant [4, 5, 17]. GS is typically less severe than BS and can have overlapping phenotypes with BS type 3 that can be difficult to distinguish without molecular diagnosis. Therefore, DNA sequence analysis is important for establishing the correct diagnosis in patients with features of BS and GS. Options for genetic analysis include custom gene panels, which are economic but limited in scope and fail to identify novel candidates; whole-exome sequencing enabling sequencing of protein coding regions that harbor approximately 85% of mutations with effects on disease-related traits but fails to identify structural variants, copy number variants, and noncoding variants; and whole-genome sequencing (WGS), which is the most comprehensive, but has higher cost, variant analysis, and data storage implications [21, 22], but that may be the only option if custom gene panels and/or whole-exome sequencing fail to identify a causative variant.

**Table 1.** Genetic basis of types of Bartter syndrome and Gitelman syndrome

Disorder	MIM^*a*^	Gene	Protein	Inheritance
BS type 1	601678	*SLC12A1*	NKCC2	MAR
BS type 2	241200	*KCNJ1*	K_ir_1.1	MAR
BS type 3	607364	*CLCNKB*	CLC-Kb	MAR
BS type 4A	602522	*BSND*	Barttin	MAR
BS type 4B	613090	*CLCNKA* and *CLCNKB*	CLC-Ka CLC-Kb	DAR
BS type 5	300971	*MAGED2*	MAGE-D2 antigen	XR
BS in association with ADH	601198	*CASR*	CASR	MAD
GS	263800	*SLC12A3*	NCCT	MAR
GS-like	601678	*KCNJ10*, *FXYD2*, and *HNF1B*	K_ir_4.1, NaK ATPase subunit gamma, HNF1B	MAR MAD MAD

Abbreviations: ADH, autosomal dominant hypocalcemia; BS, Bartter syndrome; BSND, Barttin CASR, calcium-sensing receptor; CLC-Ka, voltage-gated chloride channel-Ka; CLC-Kb, voltage-gated chloride channel-Kb; CLCNK-type accessory subunit-beta; CLCNKA, chloride channel, kidney-A; CLCNKB, chloride channel, kidney-B; DAR, digenic autosomal recessive; FXYD2, FXYD-domain containing ion transport regulator-2; GS, Gitelman syndrome; HNF1B, hepatocyte nuclear factor-1 homeobox-B; KCNJ1, potassium channel, inwardly rectifying, subfamily-J, member-1; KCNJ10, potassium channel, inwardly rectifying, subfamily-J, member-10; Kir1.1, ATP-dependent potassium channel; also referred to as renal outer medullary channel; Kir4.1, ATP-sensitive inward rectifier potassium channel 10; MAD, monogenic autosomal dominant; MAGED2, melanoma-associated antigen family member-D2; MAR, monogenic autosomal recessive; MIM, Mendelian Inheritance in Man; NaK, sodium/potassium)-transporting ATPase subunit-gamma; NCCT, thiazide-sensitive Na-Cl cotransporter; NKCC2, sodium-potassium-2 chloride cotransporter; SLC12A1, solute carrier family 12 member-1; SLC12A3, solute carrier family 12 member-3; XR, X-linked recessive.

^*a*^All entries within Online Mendelian Inheritance in Man (OMIM: OMIM.org) are given a unique and stable MIM number.

We had the opportunity to follow up one of the first reported cases of a salt-wasting tubulopathy [23] and provide her long-term outcome. Her clinical features overlapped with those of BS and GS, and because she had not undergone genetic analysis, we undertook WGS. We chose WGS because it is the most comprehensive sequencing method that enables parallel assessment of the coding and noncoding regions of the 11 known BS- and GS-associated genes, and also provides the opportunity to identify potential pathogenic variants in genes previously not known to be involved in the etiology of BS and GS.

## Materials and Methods

### Ethical Considerations

Informed consent and leukocyte DNA samples were obtained from the proband and unrelated healthy individuals using protocols approved by Multicentre Research Ethics Committee (UK) (No. MREC/02/2/93) and local and national ethics committees (Australia).

### Genome Sequencing and Variant Confirmation

Leukocyte DNA was extracted from venous blood (Gentra Puregene blood kit, Qiagen) and assessed for integrity by agarose gel electrophoresis. WGS was performed on an Illumina HiSeqX machine, reads mapped to hg19 and variants called with GATK HaplotypeCaller v3.4 and filtered based on variant quality, population allele frequency, and effect on encoded protein [24]. Variants were confirmed by DNA Sanger sequence analysis using polymerase chain reaction products generated using *CLCNKB* forward (5’-AAATCCTGCCCGGCCACTTA-3’) and reverse (5’-AGTGTTATAGGGAAGTGCCCCC-3’) primers (Life Technologies); the BigDye Terminator v3.1 Cycle Sequencing Kit (Life Technologies); and automated detection system (ABI3730 Automated capillary sequencer; Applied Biosystems). Further validation was performed by *AvaII* (New England Biolabs) restriction endonuclease digest analysis of polymerase chain reaction products. The frequency of the *CLCNKB* variant was assessed in public databases dbSNP and gnomAD v2.1.1.

### Bioinformatic Analysis of Homozygosity

Possible continuous regions of homozygosity (ROH) were detected in each autosome based on a hidden Markov model and the genotype likelihoods as implemented in bcftools roh v1.1 [25] using allele frequencies and genetic maps from the 1000G phase3 data set [24].

## Results

### Patient and Clinical Findings

A 10-year-old girl, whose parents were reportedly nonconsanguineous and came from Nisyros in the Greek Dodecanese island group, presented with 2 years of repeated episodes of cramps in her hands and feet with Trousseau and Chvostek signs; and abdominal pain associated with intercurrent viral infections and sometimes eating licorice, which can result in apparent mineralocorticoid excess [23, 26, 27]. Physical examination was normal with blood pressure 110/70. Serum biochemical results indicated hypokalemia with hypochloremic metabolic alkalosis, in association with hyponatremia, mild hypercalcemia, and normomagnesemia (Table 2) [23]. However, she subsequently developed hypocalciuria and hypomagnesemia (0.5 mmol/L). A renal biopsy was reported as normal with no evidence of juxtaglomerular hyperplasia, which is characteristic of BS [1, 28]. GS was diagnosed based on clinical features and she was treated with nonsteroidal anti-inflammatory drugs and continued oral potassium and magnesium supplementation [26].

**Table 2.** Serum clinical biochemistry of proband

	Aged 10 y pretreatment	Normal range in children	Aged 68 y on treatment	Normal range in adults
Sodium, mmol/L	129-144	133-144	143	135-145
Potassium, mmol/L	1.9-3.0	3.5-5.3	3.8	3.5-5.5
Chloride, mmol/L	84-97	98-111	96	95-110
Bicarbonate, mmol/L	29-38	19-28	33	20-32
Calcium corrected for albumin, mmol/L	2.75	2.10-2.56	2.59	2.15-2.55
Phosphate, mmol/L	2.9	0.6-1.9	0.85	0.8-1.5
Magnesium, mmol/L	0.98	0.64-1.09	0.77	0.7-1.05
Creatinine, μmol/L			119	45-85
Urea, mmol/L	10.4	1.6-6.0	10.9	3.0-8.5
Albumin, g/L			44	33-44
Parathyroid hormone, pmol/L			12.5	1.6-6.9
Vitamin D, nmol/L			83	50-250
Cholesterol, mmol/L	6.2-7.2	2.8-6.0	6.7	3.5-5.5
Triglycerides, mmol/L	> 3.75	0.4-2.1	4.7	< 1.5
Serum pH	7.45	7.35-7.45		
Glomerular filtration rate, mL/min/1.7 m^2^	135	85-150	41	90-120
Aldosterone, upright, pmol/L			> 2770	100-950
Renin, upright, mU/L			480	3.3-41
Aldosterone/renin ratio			> 6	< 70

She remained relatively asymptomatic with no manifestations of pseudogout or ectopic calcification, but had 1 miscarriage following appendicitis, followed by 2 uneventful pregnancies [26]. She underwent partial gastrectomy at age 34 years for a large gastric ulcer secondary to nonsteroidal anti-inflammatory use. At age 55 years she had developed chronic renal impairment, and a renal biopsy demonstrated moderate parenchymal injury consisting of tubular atrophy and interstitial scarring. Furthermore, she developed: type 2 diabetes mellitus; secondary hyperparathyroidism, osteoporosis, and vitamin D deficiency; glucose-6-phosphate dehydrogenase (G6PD) deficiency with hemolytic anemia from Pyridium (phenazopyridine hydrochloride); poor bladder control managed by a pacemaker; melanosis coli with colonic polyps; and depression.

**Figure 1.**
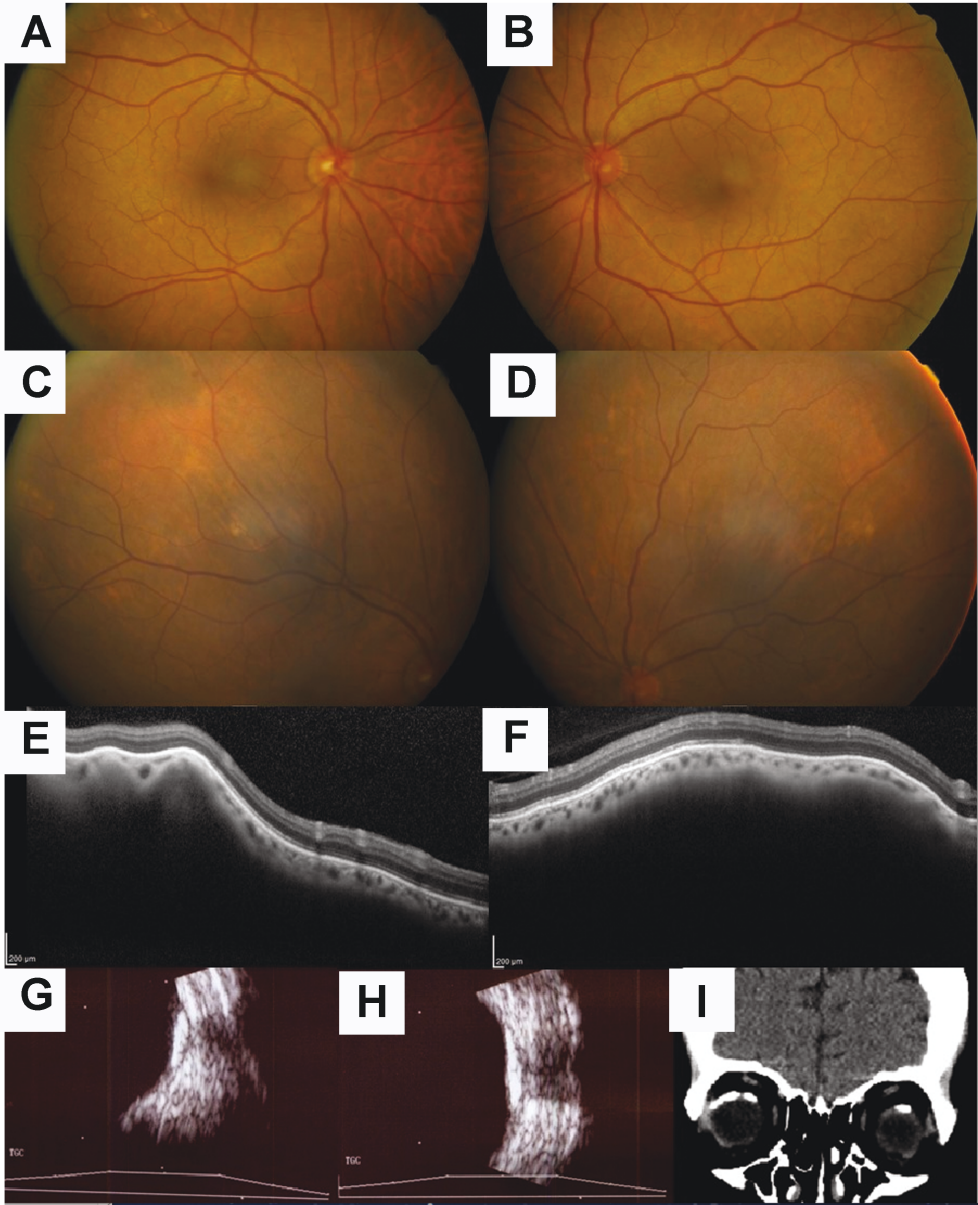
A to D, Ocular examination demonstrating unremarkable posterior poles of the A, right, and B, left, eyes, with yellow deposits superior to the vascular arcades in the A, right, and D, left, eyes. E and F, Optical coherence tomographic scanning through the deposits demonstrates choroidal elevation with overlying normal retina and focal pattern in the E, right eye, and diffuse pattern in the F, left eye. G and H, B-scan ultrasound demonstrates echogenic foci with posterior shadowing in the G, right, and H, left, eyes. I, Coronal computed tomography brain scanning demonstrates sclerochoroidal calcification in both eyes.

The patient complained of sore eyes without visual symptoms, and was referred for ophthalmic examination at age 66 years and again at age 68 years, after which scleral calcification was noted incidentally on computed tomography brain scanning. On examination, visual acuity was 6/7.5 in each eye. Anterior segments were unremarkable, with no cataracts. Fundoscopy demonstrated focal yellow choroidal deposits superior to the arcades in both eyes (Fig. 1). Furthermore, optical coherence tomography, B-scan ultrasound, and computed tomography scanning demonstrated striking sclerochoroidal calcification (SCC) calcification in both eyes (see Fig. 1), which has been associated with both BS and GS [29, 30].

At age 68 years the patient weighed 54 kg, blood pressure was 135/80 with no postural drop, and pulse was 60/minute and regular. Examination was unremarkable with no peripheral edema. Biochemical results indicated elevated serum aldosterone and renin concentrations (see Table 2). Imaging of the parathyroids and thyroid revealed no abnormalities. Her medications included potassium chloride extended-release 2.4 g twice a day orally (123 mmol/24 hours), magnesium 480 mg three times a day orally, sodium bicarbonate 100 mg twice a day orally, amiloride 10 mg twice a day orally, metformin 1 g twice a day orally, rosuvastatin 40 mg orally daily, iron polymaltose 100 mg IM, ostelin 1 daily (vitamin D and/or calcium), and denosumab 60 mg subcutaneous every 6 months. SCC was being managed conservatively, and dry eye syndrome with lid hygiene, hypromellose 3mg/g, and carbomer-980 2.2mg/g eye gel. She was also recommended to cease smoking.

At age 72 years, her weight has increased to 63 kg with a healthy body mass index of 23.0. Her serum cholesterol, high-density lipoprotein cholesterol (HDLC), low-density lipoprotein cholesterol, cholesterol/HDLC, and non-HDLC levels were within the normal range, whereas her serum triglycerides remained high at 2.0 mmol/L (normal range < 1.5 mmol/L). It is unlikely that the type 2 diabetes mellitus and dyslipidemia are related to the salt-wasting condition, although diabetic patients can develop a range of electrolyte disorders that had occurred in this patient and included hyponatremia, hypokalemia, hypercalcemia, hypocalcemia, hypomagnesemia, and hypophosphatemia [31].

### Genetic Analysis

**Figure 2.**
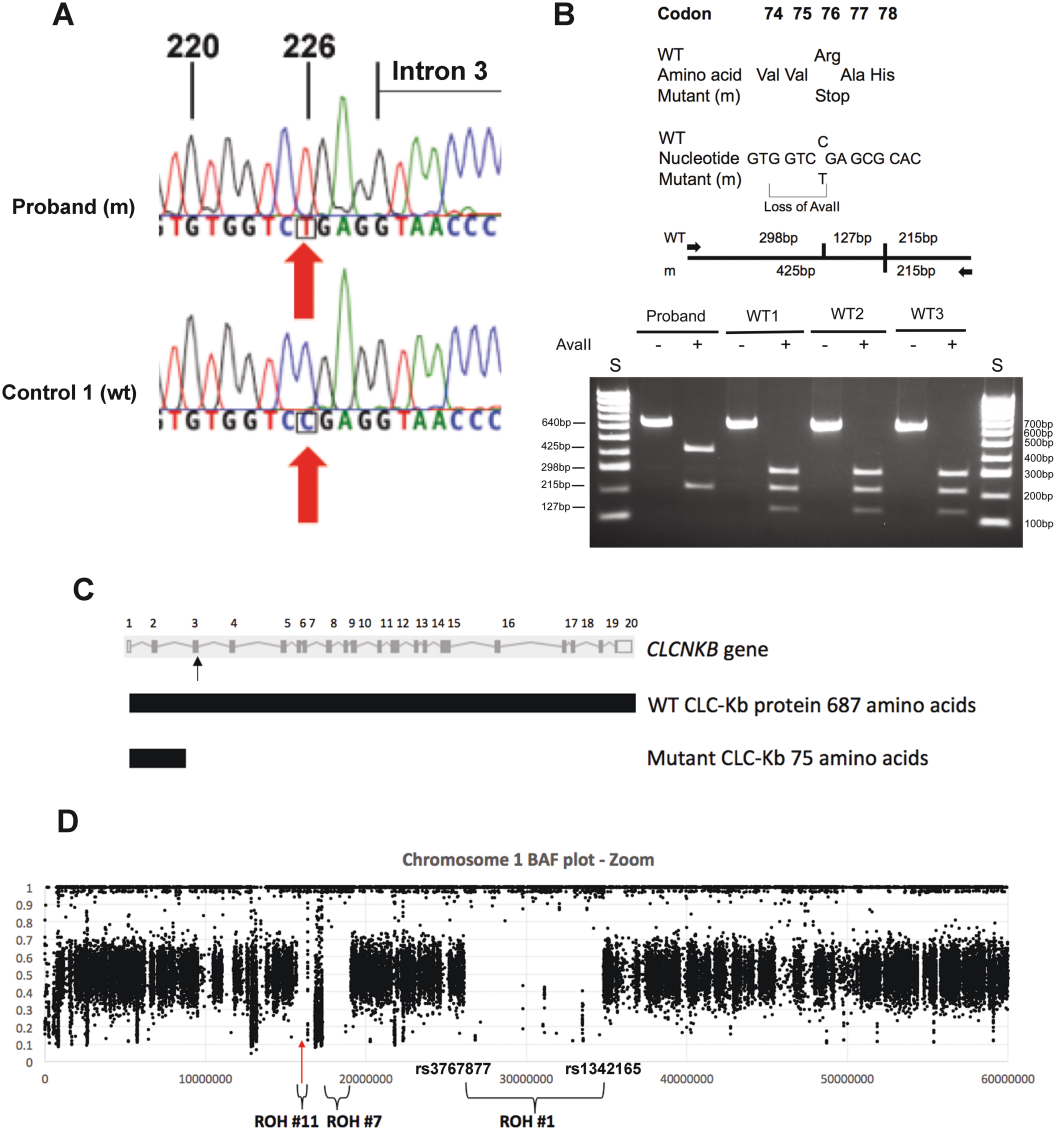
A, DNA sequence analysis showing c.226C > T (arrowed) within exon 3 of *CLCNKB* (numbering begins from ATG). The DNA sequences chromatograms show that the affected proband is homozygous T/T, while the unrelated wild-type (WT) control is homozygous C/C. B, The *CLCNKB* c.226C > T substitution is predicted to lead to a nonsense mutation of Arg, encoded by CGA, to Stop, encoded by TGA, at codon 76 and result in the loss of an *AvaII* restriction endonuclease (RE) site (G/GWC/C where W is an A or T). Restriction maps show that the *AvaII* digest would result in 3 products of 298 bp, 215 bp, and 127 bp, for the WT, and 2 products of 425 bp and 215 bp for the mutant (m). RE digest of *CLCNKB* exon 3 polymerase chain reaction products demonstrating that the proband is homozygous for m alleles, and 3 unrelated WT control individuals are homozygous for WT alleles. S, size marker. C, The *CLCNKB* gene (RefSeq NM_000085.5) contains 20 coding exons (upper panel). Exons are numbered above with the ATG start codon located in exon 2, the TGA stop codon located in exon 20, and the c.226C > T variant located in exon 3 (arrowed). The full-length WT CLC-Kb protein contains 687 amino acids (middle panel). The homozygous *CLCNKB* c.226C > T nonsense mutation identified in the patient with Bartter syndrome (BS) type 3 is predicted to cause a premature stop at codon 76 and thus loss of 612 amino acids (~90% of the protein, shown to scale) from the C-terminal end of the mutant CLC-Kb protein (lower panel). D, Allelic ratio (B-allele frequency [BAF]) plot of chromosome 1 generated from high-confidence reference single-nucleotide variant (SNV; formerly single-nucleotide polymorphism [SNP]) cluster ID (rsID) SNVs with 30× to 60× coverage. An 8.7-Mb region of homozygosity (ROH) (ROH #1) on chromosome 1p34.3-p36.11 is labeled with the flanking heterozygous SNPs rs3767877 and rs1342165, along with ROH of 1.8 Mb and 1.1 Mb (ROH #7 and ROH #11, respectively). The approximate position of the p.Arg76Ter variant in *CLCNKB*, located within ROH #11, is indicated by a red arrow. The presence of these ROH together with others [39, 40] suggest that the parents, who are deceased and from whom DNA samples are not available, are likely heterozygous for the *CLCNKB* mutation.

WGS analysis of leukocyte DNA from the patient revealed an absence of abnormalities in *SLC12A3*, *KCNJ10*, *FXYD2*, and *HNF1B*, which are reported to be associated with GS, and in *KCNJ1* or *BSND*, which are associated with BS type 2 and BS type 4A, respectively (see Table 1). Moreover, variants with an allele frequency (AF) of 16% to greater than 50% were identified in genes associated with BS types 1, 4B, 5, and ADH, thereby indicating that these commonly occurring variants were unlikely to be the cause of the disease in the patient. Thus, the variants with an AF greater than 50% in Europeans included *SLC12A1* (n = 2), which is associated with BS type 1; *CLCNKA* (n = 3), which is associated with BS type 4B; *CLCNKB* (n = 5), which is associated with BS type 4B; and *CASR* (n = 2), which is associated with ADH. A *MAGED2* variant with an AF of 16% in Europeans, which is associated with BS type 5, was also found. In contrast, WGS analysis identified a homozygous C-to-T transition rare (AF < 1%) variant at nucleotide c.226 in exon 3 of *CLCNKB* (NM_000085.5), located on chromosome 1p36.13. The C-to-T transition, which was confirmed by DNA Sanger sequence analysis (Fig. 2A), predicted the occurrence of a nonsense mutation (p.Arg76Ter) of the CLC-Kb protein and a loss of an *AvaII* restriction endonuclease site (Fig. 2B). Subsequent restriction endonuclease digest analysis confirmed the presence of the homozygous *CLCNKB* c.226C > T variant in the proband while 3 unrelated wild-type controls had only wild-type alleles (see Fig. 2B). Furthermore, this *CLCNKB* variant (c.226C > T), which is present only as a rare, heterozygous variant with allele frequencies of 0.00003 and 0.00001 in dbSNP (rs370985865) (n = 17 950 individuals) and gnomAD v2.1.1 (n = 125 563 individuals), respectively, was the sole candidate variant fitting with an autosomal recessive mode of inheritance. Thus, the homozygous occurrence of this rare *CLCNKB* c.226C > T variant in the patient, and its absence as a homozygote in more than 140 000 individuals in dbSNP and gnomAD v2.1.1, supported its pathogenic role in causing the disease of BS type 3 in the patient.

The patient was sequenced as a singleton because the parents were not available, and so phasing of variants to confirm compound heterozygosity was not possible. In addition, an examination for genes harboring at least 2 high-confidence heterozygous variants with moderate in silico support, using a Combined Annotation Dependent Deletion (CADD) score of greater than 20, which is typical of the top 1% deleterious variants in the human genome, identified only a single gene, *HMCN1* (Hemicentin-1; NM_031935.3), with 2 rare variants that were both missense changes p.(Q1174E;P2332R) with an AF less than 1%. However, *HMCN1* is reported to be associated with age-related macular degeneration and therefore does not represent a strong candidate in comparison to the known pathogenic p.Arg76Ter variant in *CLCNKB*. Thus, based on the American College of Medical Genetic and Genomics and the Association for Molecular Pathology guidelines [32], the *CLCNKB* c.226C > T (p.Arg76Ter) nonsense variant would be placed in the pathogenic, very strong 1 (PVS1) category. The PVS1 category includes null variants (nonsense, frameshift, canonical ± 1 or 2 splice sites, initiation codon, single or multiexon deletion) in a gene whose loss of function is a known mechanism of the disease, and this is consistent with a pathogenic role for the *CLCNKB* c.226C > T (p.Arg76Ter) nonsense variant in BS type 3, as it is predicted to result in loss of approximately 90% of the protein (Fig. 2C), with likely loss of its function in facilitating the flow of chloride currents through the channel, which is a known mechanism for the disease. Moreover, the *CLCNKB* c.226C > T variant has also been reported i) as a homozygous p.Arg76Ter mutation in 2 other unrelated patients with BS type 3; and ii) in 3 unrelated patients with BS type 3 as compound heterozygous mutations with p.Val170Met, p.Trp610Ter, and a c.1228-1G > A mutation involving the splice acceptor site at the boundary of *CLCNKB* intron 12 and exon 13, which is predicted to result in an in-frame insertion of 30 amino acids [33-36]. In addition, the homozygous *CLCNKB* p.Arg76Ter variant has been reported in ClinVar (ID975076.2) to be associated with familial epilepsy, hypocalciuria, and proteinuria, although plasma magnesium and calcium concentrations were not reported, and hypomagnesemia and hypocalcemia, associated with GS and BS, are known metabolic causes of seizures [37].

The *CLCNKB* c.226C > T transition, involves a CpG dinucleotide, and it is possible this transition may have involved deamination of a methylated cytosine (5-methylcytosine), resulting in formation of a thymine [38]. Thus, occurrence of the homozygous *CLCNKB* mutation in the patient may be due to 2 mutations arising de novo via deamination of 5-methylcytosines on independent haplotypes; or 1 mutation arising de novo with the other being inherited from a parent; or both mutations being inherited from heterozygote parents. We considered this latter possibility of parental heterozygosity for the *CLCNKB* p.Arg76Ter variant most likely because the parents, although reportedly nonconsanguineous, came from the Greek island of Nisyros with fewer than 1500 inhabitants, thereby increasing the likelihood of sharing a common ancestor. Since DNA from both deceased parents was unavailable, we analyzed WGS data from the proband for occurrence of genome-wide ROH, and also near the *CLCNKB* locus. This revealed 13 ROH greater than 1 Mb [39] and 3 of these, including the largest observed ROH comprising 8.7 Mb, were located on chromosome 1p34.3 to p36.21 and in the vicinity of *CLCNKB* (Fig. 2D). Another 5.1-Mb ROH was found on chromosome 6 [39, 40]. ROH greater than 4 Mb are uncommon in demonstrably outbred individuals [41], and our findings of 2 ROH greater than 4 Mb suggest a degree of unreported parental consanguinity due to a shared ancestor, which is not surprising given the small population of their island origin. Thus, distant relatedness of the parents with heterozygosity of the *CLCNKB* p.Arg76Ter mutation appears a plausible explanation for the homozygous mutation.

## Discussion

The diagnosis of GS, as the cause of the salt-wasting tubulopathy in this patient approximately 60 years ago, led to her becoming one of the first individuals described with this condition [23, 26]. However, as a result of our analysis that revealed a homozygous nonsense mutation (c.226C > T; p.Arg76Ter) in *CLCNKB*, a revised diagnosis of BS type 3 is warranted based on current classification.

The parents of the proband are reportedly nonconsanguineous. However, they originate from a Greek island with fewer than 1500 inhabitants, and may share a common ancestor who had the *CLCNKB* mutation. ROH tracts greater than 4 Mb are rare in demonstrably outbred individuals [41], and studies of consanguineous families have suggested that pathogenic homozygous variants are overrepresented within the 10 largest ROH tracts in a genome of a child with related parents [42]. Thus, identification of an 8.7-Mb ROH on chromosome 1 proximal to a 1.1-Mb ROH containing *CLCNKB*, and another 5.1-Mb ROH on chromosome 6 is consistent with the suggestion the patient’s parents are distantly related. Thus, the *CLCKNB* p.Arg76Ter may be more common in the Grecian Dodecanese group and lead to a higher incidence of BS type 3.

The original report of BS described 2 patients with hypokalemic alkalosis associated with hyperreninemic hyperaldosteronism and juxtaglomerular hyperplasia, but normal blood pressure [1]. Symptoms included failure to thrive, severe polyuria, and polydipsia due to a renal concentrating defect [1]. Subsequently, in another patient cohort, a second nephropathy was identified comprising loss of renal magnesium, intermittent episodes of tetany, normal urinary concentration, minimal involvement of the renin-angiotensin system, and an unusually low urinary calcium, that was termed *GS* to distinguish it from BS [2, 26]. However, as more cases of GS and BS type 3 (classic BS) have been reported, it is clear there is considerable overlap between these conditions [4, 5, 43, 44]. For example, hypomagnesemia and hypocalciuria, once considered pathognomonic for GS, have since been reported as features of BS [45]. Furthermore, while magnesium and calcium concentration in blood and urine were not reported in Bartter’s original description, tetany, carpopedal spasms, and positive Chvostek signs, which are symptoms of hypomagnesemia, were present in one of Bartter’s index cases, although these features could also be attributed to hypokalemia [46].

Thus, our patient has some phenotypic features more commonly associated with GS, namely low urinary calcium excretion, and other features such as renal failure and tubulointerstitial atrophy and interstitial fibrosis, which are uncommon in GS but more prevalent in BS type 3. Furthermore, similar to the clinical progression in our patient, a report of a mixed BS-GS phenotype in 3 unrelated patients described the coexistence of hypomagnesemia and hypocalciuria that was absent at presentation but subsequently detected, reflecting a transition from BS type 3 to GS [43].

These phenotypic overlaps may indicate physiological cooperation of the apical NCCT and basolateral CLC-Kb for salt reabsorption in the distal convoluted tubule (DCT). Since ion transport mechanisms are coupled to each other, loss-of-function mutations affecting one element of transepithelial transport may lead to the breakdown of absorption in affected epithelial cells. Coexistence of hypomagnesaemia and hypocalciuria in GS, and less frequently in BS type 3, suggests that the dissociation of renal calcium and magnesium handling may not always be caused by NCCT dysfunction as originally thought, but possibly because of a more general impaired transcellular salt reabsorption in the DCT involving CLC-Kb [3].

The current terminology of BS subgroups is based on the chronological order of discovery rather than their pathophysiological basis and clinical presentation. Our data support the revised classification of salt-losing tubulopathies proposed by Seyberth [3], in which milder forms of BS type 3 and GS are grouped into a single disease category distinct from more severe forms of BS. This classification thus reflects differences in DCT dysfunction (GS and BS type 3), loop disorders (BS type 1 and 2), and compound disorders (BS type 4A and 4B).

SCC, associated both with BS and GS [29, 30], is not associated with progressive visual loss, but is associated with disturbed calcium metabolism. Systemic calcification, hypomagnesemia, and chronic kidney disease, which are symptoms found in this patient, have been reported to be associated with long-term use of proton pump inhibitors [47-49]; however, this patient had not been treated with proton pump inhibitors in the preceding 10 years before SCC was detected. Therefore, genetic analysis and identification of a causative mutation were useful in excluding salt-losing conditions that occur secondary to nongenetic causes.

In summary, GS and BS type 3 have overlapping phenotypes making diagnosis based on phenotypic data alone challenging. Indeed, a recent study simultaneously sequencing 37 genes in 174 children with BS or GS revealed a discrepancy in clinical and genetic diagnosis in 10 children, with 3 cases of clinically diagnosed GS revised to BS type 3 following genetic identification of a *CLCNKB* mutation [11]. Molecular diagnosis is therefore valuable in diagnosing disorders with overlapping clinical and laboratory manifestations.

## Data Availability

The data that support the findings of the study are available from the corresponding author on reasonable request.

## Abbreviations

ADH: autosomal dominant hypocalcemia
AF: allele frequency
BS: Bartter syndrome
DCT: distal convoluted tubule
GS: Gitelman syndrome
HDLC: high-density lipoprotein cholesterol
NCCT: thiazide-sensitive Na-Cl cotransporter
ROH: region of homozygosity
SCC: sclerochoroidal calcification
WGS: whole-genome sequencing

## References

[1] Bartter FC , Pronove P, Gill JR, MacCardle RC. Hyperplasia of the juxtaglomerular complex with hyperaldosteronism and hypokalemic alkalosis. A new syndrome. Am J Med. 1962;33:811-828. 13969763 10.1016/0002-9343(62)90214-0

[2] Gitelman HJ , Graham JB, Welt LG. A new familial disorder characterized by hypokalemia and hypomagnesemia. Trans Assoc Am Physicians. 1966;79:221-235. 5929460

[3] Seyberth HW . An improved terminology and classification of Bartter-like syndromes. Nat Clin Pract Nephrol. 2008;4(10):560-567. 18695706 10.1038/ncpneph0912

[4] Besouw MTP , Kleta R, Bockenhauer D. Bartter and Gitelman syndromes: questions of class. Pediatr Nephrol. 2020;35(10):1815-1824. 31664557 10.1007/s00467-019-04371-y PMC7501116

[5] Nozu K , Yamamura T, Horinouchi T, et al Inherited salt-losing tubulopathy: an old condition but a new category of tubulopathy. Pediatr Int. 2020;62(4):428-437. 31830341 10.1111/ped.14089

[6] Konrad M , Vollmer M, Lemmink HH, et al Mutations in the chloride channel gene CLCNKB as a cause of classic Bartter syndrome. J Am Soc Nephrol. 2000;11(8):1449-1459. 10906158 10.1681/ASN.V1181449

[7] Cruz AJ , Castro A. Gitelman or Bartter type 3 syndrome? A case of distal convoluted tubulopathy caused by CLCNKB gene mutation. BMJ Case Rep. 2013;2013:bcr2012007929. 10.1136/bcr-2012-007929 PMC3604527 23345488

[8] Konrad M , Weber S. Recent advances in molecular genetics of hereditary magnesium-losing disorders. J Am Soc Nephrol. 2003;14(1):249-260. 12506158 10.1097/01.asn.0000049161.60740.ce

[9] Laghmani K , Beck BB, Yang SS, et al Polyhydramnios, transient antenatal Bartter’s syndrome, and MAGED2 mutations. N Engl J Med. 2016;374(19):1853-1863. 27120771 10.1056/NEJMoa1507629

[10] Legrand A , Treard C, Roncelin I, et al Prevalence of novel *MAGED2* mutations in antenatal Bartter syndrome. Clin J Am Soc Nephrol. 2018;13(2):242-250. 29146702 10.2215/CJN.05670517 PMC5967426

[11] Ashton EJ , Legrand A, Benoit V, et al Simultaneous sequencing of 37 genes identified causative mutations in the majority of children with renal tubulopathies. Kidney Int. 2018;93(4):961-967. 29398133 10.1016/j.kint.2017.10.016

[12] Thakker RV . Molecular pathology of renal chloride channels in Dent’s disease and Bartter’s syndrome. Exp Nephrol. 2000;8(6):351-360. 11014932 10.1159/000020689

[13] Vargas-Poussou R , Dahan K, Kahila D, et al Spectrum of mutations in Gitelman syndrome. J Am Soc Nephrol. 2011;22(4):693-703. 21415153 10.1681/ASN.2010090907 PMC3065225

[14] Bockenhauer D , Feather S, Stanescu HC, et al Epilepsy, ataxia, sensorineural deafness, tubulopathy, and KCNJ10 mutations. N Engl J Med. 2009;360(19):1960-1970. 19420365 10.1056/NEJMoa0810276 PMC3398803

[15] Scholl UI , Choi M, Liu T, et al Seizures, sensorineural deafness, ataxia, mental retardation, and electrolyte imbalance (SeSAME syndrome) caused by mutations in KCNJ10. Proc Natl Acad Sci U S A. 2009;106(14):5842-5847. 19289823 10.1073/pnas.0901749106 PMC2656559

[16] Meij IC , Koenderink JB, van Bokhoven H, et al Dominant isolated renal magnesium loss is caused by misrouting of the Na(+),K(+)-ATPase gamma-subunit. Nat Genet. 2000;26(3):265-266. 11062458 10.1038/81543

[17] Downie ML , Lopez Garcia SC, Kleta R, Bockenhauer D. Inherited tubulopathies of the kidney: insights from genetics. Clin J Am Soc Nephrol. 2021;16(4):620-630. 32238367 10.2215/CJN.14481119 PMC8092065

[18] van der Made CI , Hoorn EJ, de la Faille R, et al Hypomagnesemia as first clinical manifestation of ADTKD-HNF1B: a case series and literature review. Am J Nephrol. 2015;42(1):85-90. 26340261 10.1159/000439286

[19] Adalat S , Hayes WN, Bryant WA, et al HNF1B mutations are associated with a Gitelman-like tubulopathy that develops during childhood. Kidney Int Rep. 2019;4(9):1304-1311. 31517149 10.1016/j.ekir.2019.05.019 PMC6732753

[20] Viering D , Schlingmann KP, Hureaux M, et al; Genomics England Research Consortium. Gitelman-like syndrome caused by pathogenic variants in mtDNA. J Am Soc Nephrol. 2022;33(2):305-325. 34607911 10.1681/ASN.2021050596 PMC8819995

[21] Majewski J , Schwartzentruber J, Lalonde E, Montpetit A, Jabado N. What can exome sequencing do for you? J Med Genet. 2011;48(9):580-589. 21730106 10.1136/jmedgenet-2011-100223

[22] Petersen BS , Fredrich B, Hoeppner MP, Ellinghaus D, Franke A. Opportunities and challenges of whole-genome and -exome sequencing. BMC Genet. 2017;18(1):14. 28193154 10.1186/s12863-017-0479-5 PMC5307692

[23] Cheek DB , Robinson MJ, Collins FD. The investigation of a patient with hyperlipemia, hypokalemia, and tetany. J Pediatr. 1961;59(2):200-207. 13692712 10.1016/s0022-3476(61)80080-2

[24] Stevenson M , Pagnamenta AT, Mack, HG, et al Supplementary data for “The Bartter-Gitelman Spectrum: 50-Year Follow-up With Revision of Diagnosis After Whole-Genome Sequencing.” Accessed Feb 01, 2022. https://figshare.com/articles/journal_contribution/Fifty_Year_Follow-up_of_Gitelman_Syndrome_Revision_of_Diagnosis_after_Whole_Genome_Sequencing/19102949. 10.1210/jendso/bvac079 PMC9155595 35668994

[25] Narasimhan V , Danecek P, Scally A, Xue Y, Tyler-Smith C, Durbin R. BCFtools/RoH: a hidden Markov model approach for detecting autozygosity from next-generation sequencing data. Bioinformatics. 2016;32(11):1749-1751. 26826718 10.1093/bioinformatics/btw044 PMC4892413

[26] McCredie DA , Powell HR, Rotenberg E. Familial hypokalaemia and hypomagnesaemia, Gitelman’s syndrome, report of 6 cases. Pediatr Res. 1980;14(8):977.7422402

[27] Farese RV Jr , Biglieri EG, Shackleton CH, Irony I, Gomez-Fontes R. Licorice-induced hypermineralocorticoidism. N Engl J Med. 1991;325(17):1223-1227. 1922210 10.1056/NEJM199110243251706

[28] McCredie DA . Variants of Bartter’s syndrome. Pediatr Nephrol. 1996;10(4):419-421. 8865235

[29] Sun H , Demirci H, Shields CL, Shields JA. Sclerochoroidal calcification in a patient with classic Bartter’s syndrome. Am J Ophthalmol. 2005;139(2):365-366. 15734009 10.1016/j.ajo.2004.07.054

[30] Bourcier T , Blain P, Massin P, Grünfeld JP, Gaudric A. Sclerochoroidal calcification associated with Gitelman syndrome. Am J Ophthalmol. 1999;128(6):767-768. 10612520 10.1016/s0002-9394(99)00277-9

[31] Liamis G , Liberopoulos E, Barkas F, Elisaf M. Diabetes mellitus and electrolyte disorders. World J Clin Cases. 2014;2(10):488-496. 25325058 10.12998/wjcc.v2.i10.488 PMC4198400

[32] Richards S , Aziz N, Bale S, et al; ACMG Laboratory Quality Assurance Committee. Standards and guidelines for the interpretation of sequence variants: a joint consensus recommendation of the American College of Medical Genetics and Genomics and the Association for Molecular Pathology. Genet Med. 2015;17(5):405-424. 25741868 10.1038/gim.2015.30 PMC4544753

[33] Connaughton DM , Kennedy C, Shril S, et al Monogenic causes of chronic kidney disease in adults. Kidney Int. 2019;95(4):914-928. 30773290 10.1016/j.kint.2018.10.031 PMC6431580

[34] Hureaux M , Ashton E, Dahan K, et al High-throughput sequencing contributes to the diagnosis of tubulopathies and familial hypercalcemia hypocalciuria in adults. Kidney Int. 2019;96(6):1408-1416. 31672324 10.1016/j.kint.2019.08.027

[35] Nozu K , Iijima K, Kanda K, et al The pharmacological characteristics of molecular-based inherited salt-losing tubulopathies. J Clin Endocrinol Metab. 2010;95(12):E511-E518. 20810575 10.1210/jc.2010-0392

[36] Seys E , Andrini O, Keck M, et al Clinical and genetic spectrum of Bartter syndrome type 3. J Am Soc Nephrol. 2017;28(8):2540-2552. 28381550 10.1681/ASN.2016101057 PMC5533235

[37] Nardone R , Brigo F, Trinka E. Acute symptomatic seizures caused by electrolyte disturbances. J Clin Neurol. 2016;12(1):21-33. 26754778 10.3988/jcn.2016.12.1.21 PMC4712283

[38] Duncan BK , Miller JH. Mutagenic deamination of cytosine residues in DNA. Nature. 1980;287(5782):560-561. 6999365 10.1038/287560a0

[39] Stevenson M , Pagnamenta AT, Mack, HG, et al Supplementary data for “The Bartter-Gitelman Spectrum: 50-Year Follow-up With Revision of Diagnosis After Whole-Genome Sequencing.” Supplementary Table 1. Figshare. Accessed Feb 01, 2022. https://figshare.com/articles/journal_contribution/Fifty_Year_Follow-up_of_Gitelman_Syndrome_Revision_of_Diagnosis_after_Whole_Genome_Sequencing/19102949. 10.1210/jendso/bvac079 PMC9155595 35668994

[40] Stevenson M , Pagnamenta AT, Mack, HG, et al Supplementary data for “The Bartter-Gitelman Spectrum: 50-Year Follow-up With Revision of Diagnosis After Whole-Genome Sequencing.” Supplementary Fig. 1. Figshare. Accessed Feb 01, 2022. https://figshare.com/articles/journal_contribution/Fifty_Year_Follow-up_of_Gitelman_Syndrome_Revision_of_Diagnosis_after_Whole_Genome_Sequencing/19102949. 10.1210/jendso/bvac079 PMC9155595 35668994

[41] McQuillan R , Leutenegger AL, Abdel-Rahman R, et al Runs of homozygosity in European populations. Am J Hum Genet. 2008;83(3):359-372. 18760389 10.1016/j.ajhg.2008.08.007 PMC2556426

[42] Wakeling MN , Laver TW, Wright CF, et al; DDD Study. Homozygosity mapping provides supporting evidence of pathogenicity in recessive Mendelian disease. Genet Med. 2019;21(4):982-986. 30279471 10.1038/s41436-018-0281-4 PMC6330071

[43] Jeck N , Konrad M, Peters M, Weber S, Bonzel KE, Seyberth HW. Mutations in the chloride channel gene, CLCNKB, leading to a mixed Bartter-Gitelman phenotype. Pediatr Res. 2000;48(6):754-758. 11102542 10.1203/00006450-200012000-00009

[44] Zelikovic I , Szargel R, Hawash A, et al A novel mutation in the chloride channel gene, CLCNKB, as a cause of Gitelman and Bartter syndromes. Kidney Int. 2003;63(1):24-32. 12472765 10.1046/j.1523-1755.2003.00730.x

[45] Rudin A , Sjögren B, Aurell M. Low urinary calcium excretion in Bartter’s syndrome. N Engl J Med. 1984;310(18):1190. 10.1056/NEJM198405033101819 6709017

[46] Ault MJ , Geiderman J. Hypokalemia as a cause of tetany. West J Med. 1992;157(1):65-67. 1413751 PMC1021913

[47] Lazarus B , Chen Y, Wilson FP, et al Proton pump inhibitor use and the risk of chronic kidney disease. JAMA Intern Med. 2016;176(2):238-246. 26752337 10.1001/jamainternmed.2015.7193 PMC4772730

[48] Fusaro M , Noale M, Tripepi G, et al Long-term proton pump inhibitor use is associated with vascular calcification in chronic kidney disease: a cross-sectional study using propensity score analysis. Drug Saf. 2013;36(8):635-642. 23670724 10.1007/s40264-013-0062-6

[49] Danziger J , William JH, Scott DJ, et al Proton-pump inhibitor use is associated with low serum magnesium concentrations. Kidney Int. 2013;83(4):692-699. 23325090 10.1038/ki.2012.452 PMC5682024

